# The Influence of Dopamine on Automatic and Controlled Semantic Activation in Parkinson's Disease

**DOI:** 10.4061/2011/157072

**Published:** 2011-11-02

**Authors:** Wendy L. Arnott, David A. Copland, Helen J. Chenery, Bruce E. Murdoch, Peter A. Silburn, Anthony J. Angwin

**Affiliations:** ^1^School of Health and Rehabilitation Sciences, University of Queensland, Brisbane, QLD 4072, Australia; ^2^Centre for Clinical Research, University of Queensland, Brisbane, QLD 4029, Australia; ^3^Faculty of Health Sciences, University of Queensland, Brisbane, QLD 4029, Australia; ^4^School of Medicine, University of Queensland, Brisbane, QLD 4072, Australia

## Abstract

Two semantic priming tasks, designed to isolate automatic and controlled semantic activation, were utilized to investigate the impact of dopamine depletion on semantic processing in Parkinson's disease (PD). Seven people with PD (tested whilst on and off levodopa medication) and seven healthy adults participated in the study. The healthy adult participants demonstrated intact automatic and controlled semantic activation. Aberrant controlled semantic activation was observed in the PD group on levodopa; however, automatic semantic activation was still evident. In contrast, automatic semantic activation was not evident in the PD group off levodopa. These results further clarify the impact of PD on semantic processing, demonstrating that dopamine depletion can cause disturbances in both automatic and controlled semantic activation.

## 1. Introduction

Studies of language processing in Parkinson's disease (PD) have provided clear evidence that even in the absence of overt dementia, semantic processing impairments may be evident in some people with the disease. In addition to widely reported impairments to verbal fluency in PD [1], impairments have been observed on tasks involving action naming [2], the judgment of semantic attributes and hierarchies [3], semantic word search [4], and semantic priming [5–8]. Despite these findings, the impact of PD on automatic versus controlled mechanisms of semantic processing is still unclear. The present study sought to utilize measures of automatic and controlled semantic priming to further elucidate the impact of dopamine depletion on these mechanisms of semantic processing in PD. 

Semantic priming refers to the faster recognition of target words that are preceded by a related prime word (e.g., apple fruit) compared to an unrelated prime word (e.g., paper fruit). Importantly, these semantic priming effects can be attributed to either automatic or controlled mechanisms. Automatic semantic priming is fast acting and refers to the automatic spreading of activation from prime to target that occurs within semantic networks [9]. Controlled priming, on the other hand, is slower acting and is typically attributed to either prelexical expectancy generation or postlexical semantic checking strategies [9]. During prelexical expectancy generation, participants use the prime word to generate an expectancy set of possible target words that are related to the prime word. Lexical decisions are subsequently faster to related target words that were included within the expectancy set. In contrast, postlexical semantic matching involves retrospectively checking for a relationship between the prime and the target in order to facilitate lexical decisions. Using this strategy, participants are biased towards providing a “yes/word” lexical decision response when there is a relationship between the prime and the target, thereby facilitating lexical decisions to related target words. There are a number of critical variables that can be manipulated within an experiment in order to dissociate between these 3 mechanisms of priming [9], namely, the stimulus onset asynchrony (SOA) between prime and target, the proportion of related to unrelated word targets (relatedness proportion, RP), and the proportion of nonword targets to unrelated targets (nonword ratio). Automatic semantic activation is more likely to occur at shorter SOAs and when the RP is low and controlled expectancy-based processes typically occur at longer SOAs and when the RP is high. In contrast, postlexical semantic matching strategies can operate irrespective of the SOA and are more likely to occur when the nonword ratio is high. When the nonword ratio is high, which is often the case under conditions of a high RP, then the correct lexical decision for targets that do not share a relationship with the prime will, in most cases, be “no/nonword”. Thus, under these conditions, participants will have substantial incentive to utilize a postlexical semantic matching strategy, responding “yes/word” when the target is related to the prime and responding “no/nonword” when the target has no relationship to the prime. 

Investigations of semantic priming in healthy adults after ingestion of levodopa or other dopamine agonists have provided strong support for a neuromodulatory influence of dopamine on semantic priming. This research has suggested that increased levels of dopamine are capable of focusing semantic associations [10–13] as well as speeding the time course of semantic activation [14]. Given the dopamine depletion in PD, therefore, alterations to semantic priming will also be expected in patients with this disease.

Semantic priming tasks have been widely used by researchers to investigate semantic processing in PD [5–8, 15–17], with some researchers observing delayed automatic semantic activation in subsets of people with PD [5, 8]. Grossman et al. [8] suggested that such impairments in automatic semantic activation may be dependent upon the magnitude of dopamine depletion and frontal-striatal dysfunction in individuals with PD such that only some people with PD may exhibit delayed automatic priming. Interestingly, Copland [7] observed impairments to controlled semantic processing in the presence of spared automatic semantic processing in people with PD. Similarly, Angwin et al. [5] found that controlled semantic processing was impaired even in PD patients who presented with no delay to automatic semantic activation. Such findings suggest that even when the extent of dopamine depletion in PD is insufficient to disrupt automatic semantic activation, disturbances to controlled semantic processing can still arise. In order to more clearly differentiate between automatic and controlled semantic processing, however, comparisons of facilitation and inhibition are required. Measures of facilitation and inhibition are critical to the investigation of automatic and controlled semantic activation, because whilst automatic semantic activation produces facilitation effects (defined as faster recognition of target words following a related prime relative to a neutral prime (e.g., XXX)), controlled semantic priming is associated with the presence of both facilitation effects and inhibition effects (defined as slower recognition of targets following an unrelated prime relative to a neutral prime) [9]. Hence, measures of both facilitation and inhibition are required in order to dissociate automatic from controlled semantic processing mechanisms.

Arnott et al. [18] obtained measures of facilitation and inhibition across a battery of semantic priming tasks that were designed to explore automatic and controlled/expectancy-based semantic processing. The authors found that automatic semantic activation was delayed in PD, as evidenced by an absence of facilitation in the PD group at a short SOA and a low RP. Such findings might indicate that the magnitude of dopamine depletion in the PD group was sufficient to induce a disruption to automatic semantic activation. The authors also found that whilst the control group demonstrated both facilitation and inhibition effects at longer SOAs and a high RP, the PD group only displayed facilitation effects. These results suggested that the PD participants were unable to develop controlled expectancy-based strategies appropriately during task performance. Whilst these findings demonstrated the potential impact of dopamine depletion in PD on both automatic and controlled priming mechanisms, the PD participants in the study were only tested whilst on levodopa. 

In order to more effectively explore how dopamine depletion can influence automatic and controlled semantic activation, it is necessary to conduct within-group comparisons of PD patients on versus off levodopa. Whilst some research has suggested that automatic semantic activation is more susceptible to disruption in PD patients off relative to on levodopa [6, 19], no studies have utilized measures of both facilitation and inhibition to investigate automatic and controlled semantic activation in PD patients off medication. Accordingly, the present research aimed to address this gap in knowledge. Measures of facilitation and inhibition were obtained across both a low and a high RP semantic priming task in order to encourage automatic and controlled/expectancy-based semantic mechanisms, respectively. A low nonword ratio together with a single-choice response mode, whereby participants only respond to word targets but not nonword targets, was also utilized in order to discourage the influence of postlexical semantic matching strategies [9]. 

 It was predicted that the control group would demonstrate controlled/expectancy-based semantic processing in the high RP task, as evidenced by the presence of both facilitation and inhibition effects. It was also predicted that the control group would demonstrate automatic semantic processing in the low RP task, as evidenced by the presence of facilitation effects only. Consistent with previous findings of impaired controlled semantic activation in PD [7, 18], it was predicted that the PD group on levodopa would demonstrate facilitation effects in the absence of an inhibition effect in both the low and high RP task, indicating an impairment in controlled semantic activation. In contrast, it was predicted that the PD group off levodopa would demonstrate impairments in both automatic and controlled semantic activation.

## 2. Method

### 2.1. Participants

Seven participants (4 male) diagnosed with idiopathic PD participated in the study whilst on and off their dopaminergic medication. The PD group had a mean age of 64.29 years (SD 7.76) and a mean education of 13.86 years (SD 4.38). With respect to their disease characteristics, the participants with PD had a mean disease duration of 6.57 years (SD 4.28), a mean age at onset of 57.71 years (SD 7.57), and a mean disease severity, as measured by Hoehn and Yahr score [20], of 2.21 (SD 0.57, range 1.5–3.0). All seven participants with PD were receiving levodopa with a mean daily dosage of 450 mg (SD 227.30). One PD participant was also receiving deprenyl and another was receiving deprenyl, and pergolide. No participants with PD were taking anticholinergic medication. 

Seven nonneurologically impaired, healthy adults (4 male) also participated in the study. This control group had a mean age of 72.43 years (SD 8.06) and a mean education of 15 years (SD 5.39). The mean age and education of the PD and control groups were not significantly different. The cognitive status of each participant was also assessed prior to the commencement of the study using the dementia rating scale (DRS) [21], with a mean score for the PD group of 139 (SD 4.40) and a mean score for the control group of 141.86 (SD 1.22). All scores were above the recommended lower boundary for normal performance, and the DRS scores were not significantly different between the two groups.

The experimental tasks in this study were administered to participants as part of a larger battery of language tasks. PD participants were tested at their residence in both an on- and an off-medication state. The on-medication testing sessions were conducted approximately 45 minutes after dosage. This time delay is consistent with peak plasma levodopa levels [22] and allowed testing to take place whilst the participant was experiencing maximum clinical benefit from the medication. Off-medication sessions were conducted after participants had been without all dopaminergic medication for 12 hours.

### 2.2. Stimuli

Experimental stimuli consisted of prime-target word pairs. Targets were either real words or pronounceable nonwords, whilst primes were either category names or the letter string “xxx”. Whilst primes could be repeated within an experiment, targets appeared only once. Twelve category names were chosen as prime words, which were as follows: vehicle, sport, occupation, weapon, furniture, flower, fruit, clothing, bird, colour, animal, and tree. For each of these categories, 12 exemplars were selected from Australian category norms [23] to serve as targets, producing a master list of 144 target words. 

Three prime conditions were created for word targets. These prime conditions consisted of a related condition, in which the prime was a category name and the prime and target word were related in meaning, an unrelated condition, in which the prime was a category name and the prime and target word were unrelated in meaning, and a neutral condition, in which the prime was the letter string, “xxx”. The neutral condition was included to serve as a baseline for calculating measures of facilitation and inhibition. For nonword targets, there were two conditions, namely, “category word prime/nonword” (W/NW) and “neutral prime/nonword” (N/NW). Targets were pseudorandomly assigned to one of the five word and nonword conditions such that for each experiment, all category primes appeared the same number of times within each prime condition and each prime condition contained the same number of exemplars from each category. Pronounceable nonwords were created by changing one to two phonemes in the category exemplars that had been assigned to the nonword conditions. 

Two stimulus lists were created, one with a low RP and one with a high RP. Each list also employed a low nonword ratio (defined as the number of word prime/nonword targets divided by the number of word prime/unrelated word targets plus word prime/nonword targets). “Low”, when referring to both the RP and the nonword ratio, was defined as 0.5 or less. In order to prevent participants from adopting a strategic response bias which could distort measures of facilitation and inhibition [24, 25], targets in each list were equally likely to follow word primes as they were to follow neutral primes. Each list contained 120 word targets and 24 (12 W/NW and 12 N/NW) nonword targets. With respect to word targets, the low RP list included 12 related, 48 unrelated, and 60 neutral trials, resulting in a low RP of 0.20 and a low nonword ratio (defined as the number of word prime/nonword targets divided by the number of word prime/unrelated word targets plus word prime/nonword targets) of 0.20. In contrast, the high RP list included 48 related, 12 unrelated, and 60 neutral trials, resulting in a high RP of 0.80 and a low nonword ratio of 0.50. All experiments were computerized using Superlab experimental laboratory software (Version 1.04) [26], and the accuracy and reaction time (accurate to 1 ms) of all participant responses were recorded automatically using an RB-400 response box [26]. 

### 2.3. Procedure

The low and high RP experiments were completed during different testing sessions held at least two weeks apart. The order in which participants completed the two tasks was varied across participants. At least two weeks after completing experiments whilst on levodopa medication, the PD participants were retested whilst off medication. To avoid fatigue during medication withdrawal, the PD participants were presented with only the first two blocks (or 96 trials) for each of the priming tasks. 

The experiment involved a visual lexical decision task and a single-choice response mode. Participants were told that they would see word pairs appearing on the computer screen and they were asked to decide as quickly and as accurately as possible whether the second word in each pair was a real word or not. If the target was a real word, participants were asked to press the response button with their index finger. No response was required for nonword targets.

**Figure 1:**
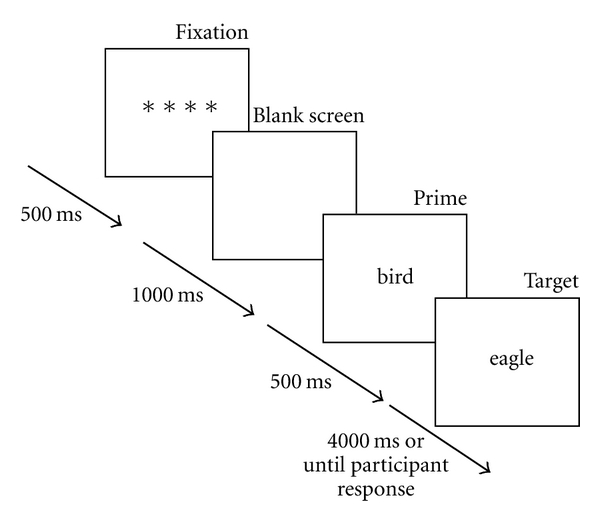
An illustration of the procedure used for a typical trial during the lexical decision task.

Figure 1 illustrates the procedure for a single trial. Each trial began with a preparatory cue, “****”, which was presented for 500 ms in the center of the computer screen. A blank screen interval of 1000 ms was then displayed, followed immediately by the prime word for 500 ms. The target was then displayed and remained on the screen for 4000 ms or until the participant pressed the response button. The next trial was automatically activated 1500 ms after the previous target disappeared from the screen. 

Participant reaction times for word targets correctly identified as words were recorded. Errors were also recorded for both “yes” responses to nonword targets and the failure to respond to word targets. Experimental stimuli were presented to subjects via three blocks of 48 trials, with a short rest break provided after each block. A practice task preceded each experiment, and participants were told that they were free to repeat the practice task until they felt confident with the procedure.

## 3. Results

### 3.1. Reaction Time Analyses

Only correct responses to word targets were analysed. Reaction times less than 200 ms and more than 1500 ms were excluded from analysis. Following removal of this data, individual participant outliers for each prime condition (defined as RTs more than 3 SDs above or below the participant mean) were also excluded from analysis. This removal of errors and outliers resulted in the removal of 3.21% of the control group's data, 4.46% of the PD on group's data, and 2.5% of the PD off group's data.  Table 1 illustrates the RTs for each group and condition.

#### 3.1.1. Controls versus PD “on” Medication

Individual participant RTs were entered into a mixed linear model analysis with participant as a random factor, Group (PD and control) as a between-subjects factor, and RP (high and low) and prime (related, unrelated, and neutral) as within-subjects factors. The analyses revealed significant main effects of RP and prime (*F*(1,3207) = 3.94, *P* = .047; and *F*(2,3207) = 13.11, *P* < .001, resp.) and significant interaction effects of group X RP, group X prime and RP X prime (*F*(1,3207) = 35.23, *P* < .001; *F*(2, 3207) = 7.71, *P* < .001; and *F*(2,3207) = 6.17, *P* = .002, resp.).

Whilst the main and interaction effects are provided for descriptive purposes, they do not test the predictions made specific in the aims of this study, namely, that patterns of facilitation and inhibition would differ between groups across the low and high RP tasks. Using separate mixed model analyses for the low and high RP tasks, therefore, facilitation effects were investigated for each group via pairwise comparisons between the related and the neutral condition (with facilitation defined as faster RTs to related targets relative to neutral targets), whilst inhibition effects were investigated via comparisons between the neutral and unrelated condition (with inhibition defined as significantly slower RTs to unrelated targets relative to neutral targets).

Analysis of the control group's data revealed significant facilitation for the low RP experiment (*t*(3207) = 2.07, *P* = .038), whilst significant facilitation and inhibition effects were evident for the high RP experiment (*t*(3207) = 6.61, *P* < .001; *t*(3207) = −2.56, *P* = .01, resp.). Analysis of the PD group's data revealed no significant facilitation or inhibition effects for the low RP experiment; however, a significant facilitation effect was evident for the high RP experiment (*t*(3207) = 2.24, *P* = .025). 

#### 3.1.2. PD “on” versus “off” Medication

Individual participant RTs were entered into a mixed linear model analysis with participant as a random factor, and medication (on and off), RP (high and low) and prime (related, unrelated, and neutral) as within subject factors. The analyses revealed significant main effects for Medication and RP (*F*(1,2679) = 14.94, *P* < .001; and *F*(1,2679) = 20.49, *P* < .001, resp.). Facilitation and inhibition effects at each RP were then analysed for the PD group off medication by way of separate planned pairwise comparisons. The analyses revealed no significant facilitation or inhibition effects for the PD group off medication.

### 3.2. Error Analyses

Word errors accounted for only 0.65% of the data and so these errors were not subjected to statistical analysis. The error rate for nonwords was higher, with an error rate of 10.12% for the control group, 11.31% for the PD on group and 7.59% for the PD off group. These nonword error rates, however, were not significantly different between the groups. The results suggest that due to the low proportion of nonword targets in the experiment, all participants may have experienced difficulties inhibiting an incorrect “yes” motor response on nonword trials. Such a response bias would not be expected to influence priming/facilitation effects, since the large error rate is only evident for the nonword trials.

## 4. Discussion

The present research sought to investigate automatic and controlled semantic processing in PD patients on and off levodopa via measures of facilitation and inhibition across a low RP and a high RP semantic priming task. The results indicated disruptions to both automatic and controlled semantic processing mechanisms in PD. The following discussion will consider the findings for each group and the potential neuromodulatory influence of dopamine on semantic processing. 

### 4.1. Control Group

As predicted, the use of a low nonword ratio and a low RP successfully isolated automatic semantic activation in the control group, as evidenced by the presence of a facilitation effect in the absence of an inhibition effect in the low RP task. Also consistent with predictions, the use of a low nonword ratio and a high RP successfully induced controlled expectancy-based semantic processing. In the high RP task, the control group demonstrated both facilitation and inhibition effects, a pattern of results that is consistent with Neely's [9] account of controlled processing. Since the use of a low nonword ratio together with a single-choice response mode in the current study would discourage the use of postlexical semantic matching strategies, the presence of facilitation and inhibition in the high RP task would be consistent with controlled expectancy-based processing. Overall, the results for the control group were consistent with predictions and offer a valid baseline against which to interpret the performance of the PD group on and off levodopa. 

### 4.2. PD Group on Levodopa

It was predicted that the PD group on levodopa would demonstrate aberrant controlled semantic processing. The results for the high RP task supported this prediction, with the PD group on levodopa showing a facilitation effect in the absence of an inhibition effect, which is consistent with automatic rather than controlled semantic processing. These results are consistent with those previously reported by Arnott et al. [18], which also revealed a pattern of facilitation dominance at SOAs of 500 ms and longer in the high RP task. 

Previous research has illustrated that aspects of controlled lexical-semantic processing are compromised in PD [7, 27]. Semantic priming research in healthy adults on levodopa has also suggested that dopamine modulates postlexical semantic matching strategies [11]. Accordingly, the present results provide further support for the notion that PD is characterized by deficits to controlled processing and further illustrate that dopamine's influence on controlled processing may extend to prelexical expectancy generation strategies. 

Arnott et al. [18] proposed that the disruption to expectancy-based processing might be due to a decrease in the signal to noise ratio of information processing. Specifically, researchers have suggested that dopamine is capable of integrating relevant information and screening out irrelevant information within neural networks [28]. Accordingly, dopamine depletion in PD can be expected to lead to a decreased signal to noise ratio, which may disrupt processing of prime and target words during performance of a semantic priming task. Arnott et al. [18] suggested that as a consequence of a reduced signal to noise ratio, people with PD fail to detect the high proportion of semantically related word pairs in the high RP task and so do not engage in the creation of expectancy lists. Indeed, deficits in PD have also been documented for other cognitive tasks involving internal, as opposed to external, strategy generation [29], suggesting that people with PD may have difficulty developing expectancies unless their attention is drawn to the semantic relationships within the task. Supporting this suggestion, Arnott et al. [18] found that PD patients were able to create expectancies appropriately during an offline semantic judgement task when they were made aware of a semantic relationship between stimulus items. Price [30] also demonstrated that problem solving deficits exhibited by PD patients on an anagram task were remediated by the provision of cues which supported the generation of appropriate strategies. In order to engage in controlled processing effectively, therefore, PD patients may require the provision of increased contextual support for the task.

Rather than an inability to detect the high RP and a subsequent failure to engage in strategic processing, it is possible that PD patients did detect the high RP but were unable to develop appropriate expectancies during performance of the task. Indeed, there is much evidence to suggest that basal ganglia dysfunction can lead to such impairments. Gold et al. [31] revealed impaired lexical-semantic strategy formation in a patient with bilateral striatocapsular infarctions, suggesting that dysfunction within dorsolateral prefrontal subcortical circuitry can lead to impairments in strategic semantic processing. In a review of literature on rule-based category learning, Price et al. [32] also illustrated that rule generation, maintenance, and selection can all be potentially impacted by PD and/or medication. Deficits in these processes would also be expected to lead to difficulties in the performance of expectancy based processing. Goebel et al. [33] recently demonstrated that whilst PD patients were able to develop appropriate internal strategies during a cognitive task, they took longer than control participants to develop these strategies. It is also possible, therefore, that PD patients may have been slower than controls in their recognition of the high RP and/or in their development of expectancy-based strategies. 

Whilst the results indicate a clear disturbance in the ability to generate expectancies in PD, the presence of facilitation in the high RP task suggests that automatic semantic activation is intact for the PD group. This finding appears consistent with previous findings of impaired controlled processing in the presence of spared automatic semantic activation in PD [7]. Given previous research suggesting that an increased magnitude of dopamine depletion in PD can delay automatic semantic activation [5, 6, 8], the findings of this study might also appear to suggest that the severity of dopamine depletion in this cohort of participants was not sufficient to delay automatic semantic activation. It should be noted, however, that the use of only a 500 ms SOA in the present study would not be sensitive to earlier changes in the time course of automatic semantic activation. For instance, Angwin et al. [5] previously found that whilst one subgroup of PD patients showed semantic priming at both a 250 ms and a 600 ms SOA, another subgroup of PD patients showed delayed automatic semantic activation as evidenced by an absence of priming at 250 ms SOA but the presence of priming at 600 ms SOA. Accordingly, it is possible that automatic semantic activation is still delayed in the present cohort but that the use of only a 500 ms SOA was not sensitive to delays in semantic processing.

Also worthy of note is that in the absence of prime generated expectancies, automatic semantic activation would be expected to lead to facilitation in both the low and high RP tasks. Hence, it is surprising that there was a complete absence of facilitation evident in the low RP task for the PD group on levodopa. One explanation for this result could relate to the repetition of prime words within the experiment. Specifically, although target words were only presented once, prime words were repeated a total of 6 times within both the low and high RP tasks. In addition, there were 4 times as many occurrences of related prime target pairs in the high RP task (48 related trials) relative to the low RP task (12 related trials). It is possible that the effects of prime repetition together with the larger number of related trials in the high RP task lead to significant facilitation in the high RP task only. 

Repetition priming, whereby stimuli are processed more quickly and accurately upon a second presentation relative to a first presentation, is a robust and well-recognized phenomenon. Research has demonstrated that such effects are long lasting and that the repetition priming effect accumulates as a function of the number of times a stimulus is presented [34]. It is reasonable to conclude, therefore, that participants in the present study would not only recognize repeated primes more quickly than upon first presentation of the prime but that this recognition would become progressively faster over the course of the experiment, as primes were repeated multiple times. Hutchison et al. [35] recently demonstrated that semantic priming effects in healthy adults are larger when related prime words are short and have few orthographic neighbours, which may be linked to the fact that prime words that are quickly recognized can exert more influence upon processing of the target word. Accordingly, if the repeated prime words in the present study were recognized more quickly with each subsequent repetition, and if quickly recognized primes have a larger influence upon processing of target words than more slowly recognized prime words, then facilitation effects for the related trials of the present study would become larger as the experiment progressed, because participants would recognize the primes progressively more quickly. For participants with PD, therefore, facilitation effects may have only been induced once primes were recognized quickly enough after multiple repetitions. Since there were only 12 related word pairs in the low RP task, then even when prime words begin to be recognized sufficiently quickly to induce facilitation, there might be insufficient trials left in the experiment to measure this effect. In contrast, the high RP task consisted of 48 related trials such that once primes were being recognized sufficiently quickly to induce facilitation, there was a sufficient number of trials left in the experiment to detect this effect. 

The question remains, however, as to why the control group still demonstrated facilitation in the low RP task whilst the PD group did not. In a semantic priming study on healthy adults, Copland et al. [11] proposed that increased dopamine levels following ingestion of levodopa lead to increased semantic salience and a focusing of activation within semantic networks. Copland et al. [11] suggested that these changes were consistent with dopamine's influence on the signal-to-noise ratio of information processing. Similarly, researchers have suggested that dopamine depletion in PD can lead to an opposite effect, such that prime activation may be weakened or obscured by noise in PD, increasing the susceptibility of semantic priming to disruption [5, 19]. In the present study, therefore, this reduced prime salience for participants with PD could be gradually remediated over the course of the experiment due to the repeated presentation of the prime words, subsequently leading to facilitation effects in the high RP task.

It should be noted, however, that previous research has successfully obtained priming in PD without the repetition of prime words. Most previous studies, however, have not used category priming to investigate semantic activation in PD (e.g., [5–7, 19]), and as indicated previously by Arnott et al. [18], category priming may be subject to a shorter time course of activation. Hence, category priming may be more susceptible to disruption from reductions in prime salience in PD. We acknowledge that our suggestion of an influence of prime repetition on priming/facilitation effects is only speculative at this point. In order to verify the above suggestions, additional research in a larger cohort of PD patients and with a larger number of experimental trials is required. Such research should focus on investigating whether the number of prime repetitions and/or the speed of prime recognition influence category priming as well as the priming of other semantic and associative relationships.

Overall, the results of this study add to the growing evidence that various aspects of semantic processing may be compromised in PD [2, 3, 36, 37]. The results also support previous suggestions that disruptions to the signal-to-noise ratio may influence semantic processing in PD [19], and illustrate that such changes to the signal to noise ratio can have a differential impact on automatic and controlled aspects of semantic activation. 

### 4.3. PD Group off Levodopa

It was hypothesized that further disturbances to automatic semantic activation would be evident in the PD group off levodopa relative to when on levodopa. Consistent with predictions, analysis of the data for the PD off group revealed an absence of facilitation for both the low and the high RP tasks, which contrasts with the facilitation that was evident in the high RP task when these same patients were tested on levodopa. Worthy of note is that the absence of facilitation was not associated with an increased error rate or slower RTs for PD patients off, relative to on, levodopa, and so, it cannot be attributed to slower motor responses or decreased attention to the lexical decision task during medication withdrawal. The results are, therefore, consistent with the neuromodulatory influence of dopamine on the signal-to-noise ratio. As discussed previously, prime activation may be weakened by dopamine depletion and a reduced signal-to-noise ratio in PD such that repeated prime presentations were necessary for facilitation to be obtained in the PD group on levodopa. For the PD group off levodopa, the larger magnitude of dopamine depletion would be expected to lead to a further weakening of prime activation. As a consequence, even the repeated prime word presentations are no longer sufficient to overcome the weakened prime activation, leading to an absence of automatic semantic facilitation in both the low and high RP tasks. 

An important consideration, however, is that the PD group off levodopa was only tested on the first 2 blocks of each experiment. Hence, it could be argued that the absence of facilitation for this group in the high RP task is not a result of increased dopamine depletion but is instead simply a result of fewer instances of prime repetition. A lower number of prime repetitions could have subsequently prevented prime activation levels from reaching a sufficient level to induce facilitation. In order to investigate whether this explanation could account for the results, the data for the PD group on levodopa was reanalyzed after excluding the final block of items from analysis. The results of this reanalysis revealed that the facilitation effect for the PD group on levodopa was still maintained for the high RP task. Thus, the findings for the PD group off levodopa cannot be explained by fewer prime repetitions during the off-medication testing session, but rather, it may be explained by a larger reduction in prime activation in PD patients during medication withdrawal. This finding further supports the suggestion that dopamine depletion leads to weakened prime word activation in semantic priming tasks. Previous research in healthy adults [13] as well as PD [38] has suggested that dopaminergic modulation of semantic priming is mediated by D1 receptors. Thus, stronger activation of prime words in PD patients on relative to off levodopa, as suggested by the results of the present study, may be mediated by D1 receptors. 

The results of this study also have important implications for cognitive decline in PD. Williams-Gray et al. [39] demonstrated that semantic fluency impairments were a predictor of cognitive decline in PD, which they suggested was a reflection of probable nondopaminergic cortical pathology. The results of the present study suggest that semantic impairment in PD may, in fact, also be linked to dopaminergic pathology. Such findings highlight the need for additional research into predictors of cognitive decline in PD and the investigation of the contribution that dopaminergic pathology may have toward this cognitive decline.

There were a number of limitations in the present study. Firstly, the study was limited by a small sample size of only mildly impaired PD participants, so the results should be interpreted cautiously. Further research with a larger cohort of participants is warranted in order to validate the results obtained by this study. Further research should also consider the potential significant influence of PD-related disease variables on semantic processing, as researchers have indicated that there may be an important relationship between cognitive dysfunction in PD and disease-related variables such as side of onset or motor symptoms [40]. As discussed earlier, another limitation of the present study is that only a 500 ms SOA was implemented. Specifically, the use of a shorter SOA in future research will allow for a more effective investigation of whether changes to the early time course of automatic semantic activation are evident in PD. In spite of these limitations, the results of this study provide valuable information on the impact of PD on aspects of both automatic and controlled semantic processing.

### 4.4. Conclusions

In summary, the present study employed two semantic priming tasks to investigate automatic and controlled semantic priming in PD patients on and off levodopa. The results indicated that controlled semantic activation is most sensitive to disruption in PD, suggesting that even a mild depletion of dopamine may be sufficient to prevent the formation of controlled expectancy-based semantic activation. The results also indicated disruptions to automatic semantic activation in PD, which may be exacerbated by medication withdrawal. The findings are consistent with the potential influence of frontal-striatal circuitry on both automatic and controlled semantic activation.

## Figures and Tables

**Table 1:** Mean RTs in milliseconds for the control group and the PD group “on” and “off” medication as a function of RP and prime condition.

Prime Condition	Group					
	Control		PD on		PD off	
	Low RP	High RP	Low RP	High RP	Low RP	High RP
Related	580 (105)	588 (101)	619 (184)	578 (122)	598 (156)	553 (108)
Unrelated	607 (101)	672 (198)	609 (135)	605 (158)	597 (146)	583 (138)
Neutral	605 (101)	641 (143)	613 (161)	593 (151)	598 (161)	563 (130)

Note. Standard deviations in brackets; PD: Parkinson's disease; RP: Relatedness Proportion.
